# Dimensional stability and electrochemical behaviour of ZrO_2_ incorporated electrospun PVdF-HFP based nanocomposite polymer membrane electrolyte for Li-ion capacitors

**DOI:** 10.1038/srep45390

**Published:** 2017-04-11

**Authors:** Arun Kumar Solarajan, Vignesh Murugadoss, Subramania Angaiah

**Affiliations:** 1 Electrochemical Energy Research Lab, Centre for Nanoscience and Technology, Pondicherry University, Puducherry-605 014, India

## Abstract

Different weight percentages of ZrO_2_ (0, 3, 5, 7 and 10 wt%) incorporated electrospun PVDF-HFP nanocomposite polymer membranes (esCPMs) were prepared by electrospinning technique. They were activated by soaking in 1 M LiPF_6_ containing 1:1 volume ratio of EC : DMC (ethylene carbonate:dimethyl carbonate) to get electrospun nanocomposite polymer membrane electrolytes (esCPMEs). The influence of ZrO_2_ on the physical, mechanical and electrochemical properties of esCPM was studied in detail. Finally, coin type Li-ion capacitor cell was assembled using LiCo_0.2_Mn_1.8_O_4_ as the cathode, Activated carbon as the anode and the esCPME containing 7 wt% of ZrO_2_ as the separator, which delivered a discharge capacitance of 182.5 Fg^−1^ at the current density of 1Ag^−1^ and retained 92% of its initial discharge capacitance even after 2,000 cycles. It revealed that the electrospun PVdF-HFP/ZrO_2_ based nanocomposite membrane electrolyte could be used as a good candidate for high performance Li-ion capacitors.

Polymer electrolytes are promising alternative to the conventional liquid electrolytes, as they overcome the problems caused by liquid electrolytes such as leakage that are deteriorating the long term performance of Li-ion capacitors as well as it can easily be form in any possible shapes^1^. Currently, great efforts has been made on developing nanocomposite polymer membrane electrolytes, in order to improve the ionic conductivity, mechanical strength, stiffness, dimensional stability, thermal stability, electrochemical stability and good electrode-electrolyte contact. The dispersion of nanofillers such as SiO_2_, Al_2_O_3_, TiO_2_, ZrO_2_, etc., in the polymer electrolytes have been reported to be able to prevent the crystallization/reorganization of the polymer chains by acting as solid plasticizer and promote ionic mobility and ionic dissociation through Lewis acid-base interaction between the fillers and polymer or fillers and the ionic species^2^,^3^. Among many polymer electrolytes, electrospun polymer membrane electrolytes has attracted immense interest because their three-dimensional interconnected porous structure that facilitate the high electrolyte uptake, high ionic mobility, good dimensional stability and prevent electrolyte leakage. A majority of studies on PVdF-HFP based nanocomposite polymer electrolytes were reported for lithium-ion battery applications because of their excellent ionic conductivity, electrochemical stability and mechanical strength etc., The effect of zirconia fillers on the structural, mechanical, thermal and electrochemical properties of the polymer electrolytes such as polyvinyl alcohol (PVC), poly (ethylene oxide) (PEO), etc., have been reported earlier^4^,^5^,^6^,^7^. However, there are only few reports on PVdF-HFP based polymer electrolytes for supercapacitor applications and so far no literature is available for electrospun PVdF-HFP/ ZrO_2_ nanocomposite polymer membrane electrolyte for Li-ion capacitors. Therefore, the development of electrospun PVdF-HFP/ZrO_2_ nanocomposite polymer membrane for Li-ion capacitors is of great importance.

In the present work, we have attempted to incorporate zirconia (ZrO_2_) into electrospun PVdF-HFP based membrane to enhance its thermal, mechanical and electrochemical properties. Finally, the supercapacitive performance of LiCo_0.2_Mn_1.8_O_4_//Carbon Li-ion capacitor cell assembled with the prepared electrospun PVdF-HFP/ZrO_2_ nanofibrous composite membrane containing 1 M LiPF_6_ in EC and DMC (1:1 v/v) was studied in detail.

## Results and Discussion

### Physical Characterisation

**Figure 1:**
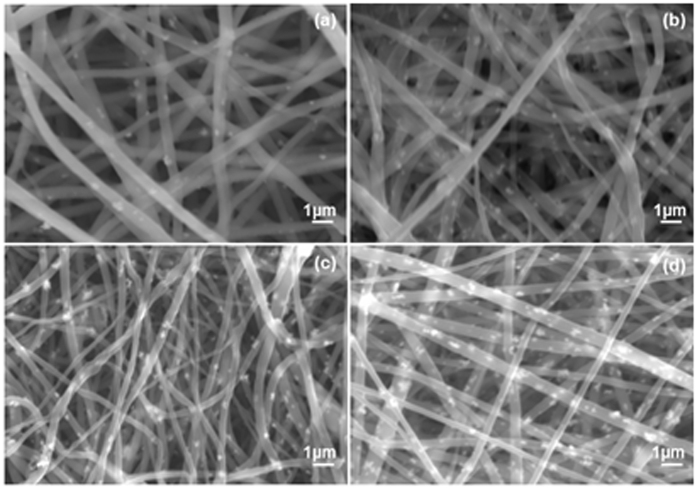
SEM images of different wt% of ZrO_2_ incorporated esCPM (**a**) 3 wt%, (**b**) 5 wt%, (**c**) 7 wt% and (**d**) 10 wt%.

Figure 1(a–d) shows SEM images of different wt% of ZrO_2_ incorporated esCPMs which exhibit three-dimensional well-interconnected network structures. The interconnected porous structure promotes the diffusion of ions and interfacial contact with the electrodes. It can be observed that the membranes consist of smooth nanofibers with an average fiber diameter of ‘400 nm. It can be seen that ZrO_2_ nanoparticles were dispersed homogeneously in PVdF-HFP matrix that may be due to good compatibility between the ZrO_2_ nanoparticles and the polymer. The smooth surface morphology represents a more amorphous phase which will cause the polymer electrolyte more flexible and the conducting ions will move freely in the polymer electrolyte. The surface the electrospun membrane became rougher beyond the addition of 7 wt% of ZrO_2_ nanoparticles. At the higher contents of ZrO_2_ over 7 wt%, the SEM morphology shows large number of ZrO_2_ aggregates, which causes blocking effect for ionic conduction.

The porosity and electrolyte uptake as a function of different weight percentages of ZrO_2_ in esCPMs are shown in Fig. S1 and their values are given in Table 1. The porosity of esCPM increased from 82.2% to 95.7% up to 7 wt% of ZrO_2_ content and then decreased on further increase in ZrO_2_ content (85.4%). The higher porosity facilitate electrolyte uptake and thus 7 wt% of ZrO_2_ incorporated esCPM has a maximum electrolyte uptake of 481%. The absorbed electrolyte is well retained even under the pressure for cell assembly and shows a low leakage of 0.15%.

**Figure 2:**
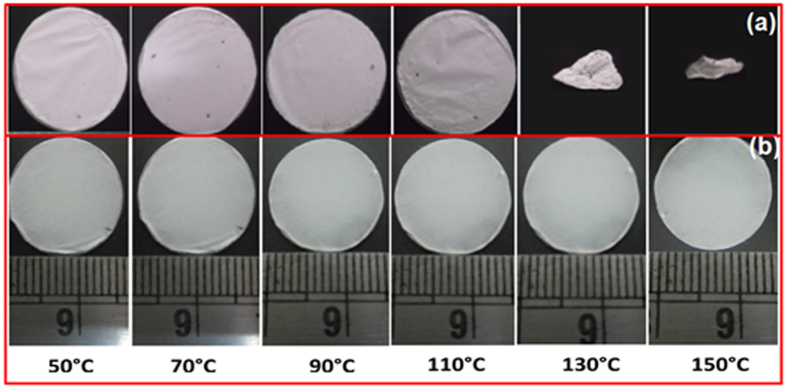
Dimensional shrinkage of (**a**) esPM and (**b**) 7 wt% of ZrO_2_ incorporated esCPM as a function of temperature.

The esCPMs must have excellent dimensional stability for a broad temperature range of operation, as the exothermic reaction take place during charge - discharge cycle of Li-ion capacitors which may cause thermal shrinkage of the polymer membrane. The thermal shrinkage of the membrane may cause short circuiting. Figure 2a,b represents the photographs of esPM and 7 wt% of ZrO_2_ incorporated esCPM after being exposed to heat treatment at various temperatures, respectively. It is observed that the esPM can withstand up to only 90 °C and starts to shrink at 110 °C. The shrinkage was found to be more than 75% at 150 °C. But, in case of the 7 wt% of ZrO_2_ incorporated esCPM there is no apparent thermal shrinkage even at 150 °C. The heat-resistant ZrO_2_ nanoparticles are believed to effectively prevent the esCPM based separator from its thermal shrinkage. This may be attributed to the interaction between the polymer chains and ZrO_2_ nanoparticles. The acidic sites on the surface of ZrO_2_ nanoparticles provide better thermal oxidation stability to host PVdF-HFP matrix. This indicates that ZrO_2_ incorporation extensively improved the dimensional stability of esCPME and thereby making it as efficient candidate for high voltage Li-ion capacitors.

**Figure 3:**
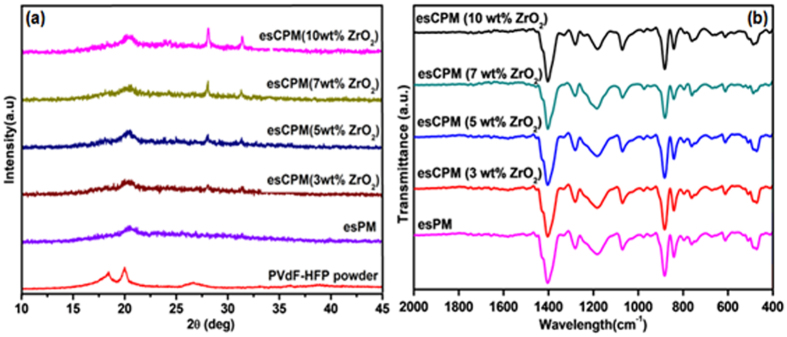
(**a**) XRD patterns of PVdF-HFP powder, esPM and different wt% of ZrO_2_ incorporated esCPMs. (**b**) FTIR spectra of different wt% of ZrO_2_ incorporated esCPMs.

Figure 3a shows the X-ray diffraction patterns of PVdF-HFP powder, esPM and esCPMs. The major peaks at 18.2° and 20.0° correspond to (100) and (020) crystalline phases of PVdF-HFP powder, respectively. The peaks at 28.12°, 31.44°, 34.25° and 35.30° appears in the XRD patterns of esCPMs are characteristics of ZrO_2_ nanoparticles. The incorporated ZrO_2_ nanoparticles in esCPMs are well intercalated in the polymer chain and hence no obvious peaks corresponding to ZrO_2_ nanoparticles are observed in X-ray diffraction analysis^8^. The addition of ZrO_2_ nanoparticles decreases the intensity of characteristic peaks of PVdF-HFP at 18.2° and 20.0°, suggesting the reduction of crystallinity in the host PVdF-HFP polymer. This leads to increase in the flexibility of polymer chain which is critical requirement to obtain high ionic conductivity for polymer membrane electrolyte for Li-ion capacitor applications^9^. Meanwhile for esPM and esCPMEs, the peak at 2θ = 18.2° indicates that the α-phase of PVdF is suppressed and only the β phase exists. The stabilization of β phase of PVdF may attribute to the spatial confinement of the polymer chains forced by the rigid nanofillers^10^. The interaction of nanofillers with polymer hinders the re-organization of polymer chain thereby stabilizes the amorphous phase of polymer electrolyte. This in turn favours intrachain and interchain hopping of ionic species and thereby enhancing the ionic conductivity. It is observed that the crystallinity does not falls continuously with the increase in wt% of ZrO_2_. This behaviour is resulting from the high concentrations of ceramic fillers, which leads to well-defined crystalline region. Above the optimum concentration of 7 wt% ZrO_2_ nanoparticles impede the mobilization of polymer chains, which is unfavourable for ion transportation.

Figure 3b shows the FTIR spectra of esPM and esCPMs. The band at 510 cm^−1^ is assigned to the CF_2_ bending vibration. The band at 1181 is due to the C-C bond of PVdF. The peak at 1402 cm^−1^ corresponds to bending vibration of the vinyl group. The vibration band of at about 1200 cm^−1^ corresponds to C-F stretching. The absorption band appeared at 882 cm^−1^ is assigned to the characteristic frequency of vinylidene group. The peak at 1071 cm^−1^ is due to -C-F_2_- stretching mode. The peak at 510 cm^−1^ and 840 cm^−1^ are due to β phase of PVdF. The peaks at 763 cm^−1^ are due to α phase of PVdF. Thus the FTIR results are consistent with XRD results. No other impurities were detected, which indicates the formation of PVDF-HFP/ZrO_2_ based nanofibrous composite membrane.

DSC thermograms of esPM and esCPMs with different wt% of ZrO_2_ nanoparticles are shown in Fig. S2. It is observed that the addition of ZrO_2_ nanoparticles slightly influence the melting temperature of PVdF-HFP polymer. The melting temperature of esPM and esCPMEs and their corresponding percentage of crystallinity are listed in Table 1. On increasing the wt% of ZrO_2_, the crystallinity decreases up to the optimum concentration of 7 wt%, which is consistent with the XRD results. The decrease in crystallinity enhanced the ion transportation and thereby prevents the ion cluster formation. Beyond the optimum concentration of 7 wt%, steric hindrance caused by both PVdF-HFP and ZrO_2_ nanoparticles induces the crystalline region which leads to the lower segmental mobility of the polymer chain.

Figure S3 shows the TGA curves of esPM and esCPMs and their corresponding parameters are listed in Table S1. It is observed that the incorporation of ZrO_2_ nanoparticles increases the decomposition temperature of the esCPMs and the percentage of weight loss decreases with the increase of ZrO_2_ content in the esCPMs. The esCPM possess decomposition temperature above 480 °C, which is higher than that of esPM that fulfill the safety requirements of Li-ion capacitors. This is attributed from the passivation layer formed by the addition of ZrO_2_ nanoparticles which act as an insulator for mass and heat transport during thermal degradation. It is well known that amorphous material have lower decomposition temperature compared with crystalline material. Hence, the decomposition temperature of the esCPM increases up to the concentration of 7 wt% and then decreased for 10 wt% of ZrO_2_ incorporated esCPM. This may be due to the aggregation of ZrO_2_ nanoparticles which affect the passivation that leads to a slight decrease in the decomposition temperature^11^. This result has good consistent with the XRD and DSC results.

**Figure 4:**
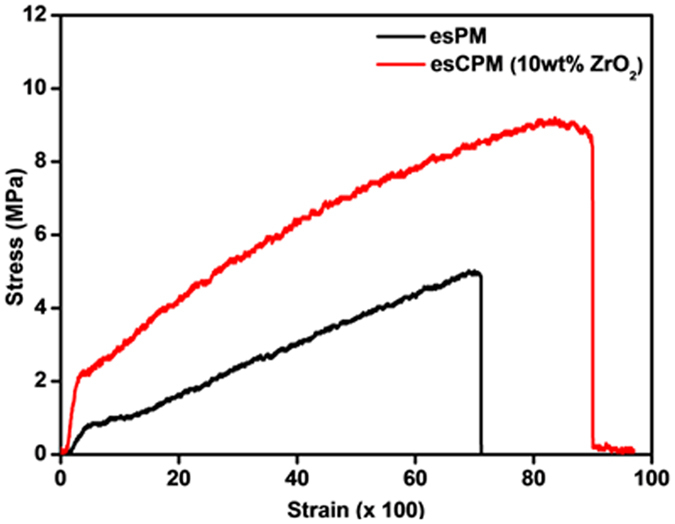
Stress-strain curves of esPM and esCPM (7 wt% ZrO_2_).

The investigation of mechanical properties of the prepared electrospun polymer membrane is significantly important, as the electrolytes for Li-ion capacitors must withstand the pressure applied during the device packaging and cycling. Generally, the higher apparent young’s modulus for the electrospun polymer membranes are due to the cross-linking of the nanofibers which act as high density point that helps to improve the dimensional stability^12^. Figure 4 shows the stress-strain curves of esPM and esCPM (7 wt %). The maximum transverse strength is 9.1 MPa for esCPM (7 wt% ZrO_2_) which is about 178% higher than that of esPM (5.1 MPa). Thus, the incorporation of ZrO_2_ improves the mechanical strength of esCPM and thereby makes it able to withstand the stress during the packaging and charge-discharge cycles.

### Electrochemical studies

**Figure 5:**
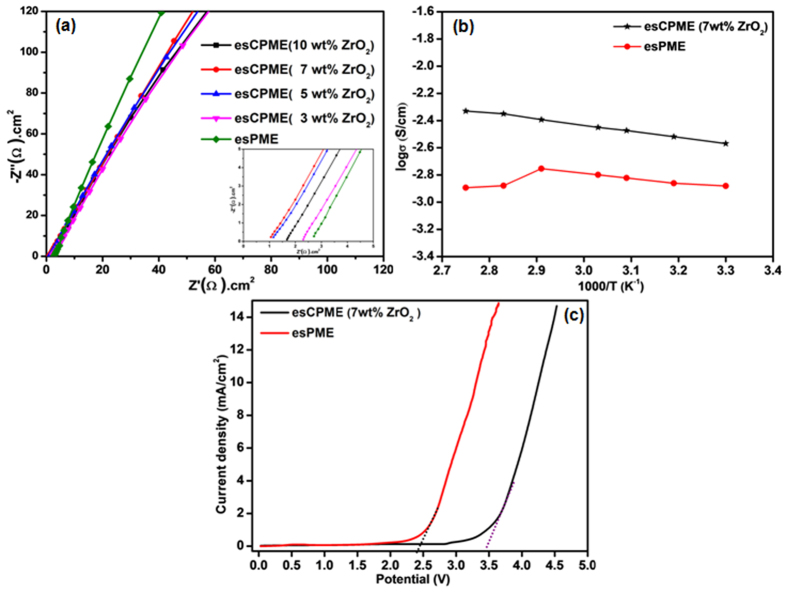
(**a**) Nyquist plots for esPME and esCPMEs. (**b**) Temperature dependence of ionic conductivity of esPME and 7 wt% of ZrO_2_ incorporated esCPME. (**c**) Linear sweep voltammetry curves of esPME and 7 wt% of ZrO_2_ incorporated esCPME.

Figure 5a shows Nyquist plots for esPME and esCPMEs. The plots show a nearly linear curve which corresponds to the lower frequency region, implying that the esPME and esCPMEs possess ideal capacitive performance and the current carriers in electrolyte are ions and the majority of the conduction is due to only by ions not by the electrons^13^. This indicates that the esCPMEs will provide better electrochemical performance and better compatibility with the electrodes. The ionic conductivities of esPME and esCPMEs were determined by AC-impedance technique at room temperature. Figure S4 shows the variation of room temperature ionic conductivity of esCPME with respect to different weight percentages of ZrO_2_ and the values are given in Table 1. The ionic conductivity increases with increase in ZrO_2_ nanoparticles up to the optimum concentration of 7 wt%. The decrease in the crystallinity of PVdF-HFP and creation of new pathways for the ion transfer created by the addition of ZrO_2_ nanoparticles attributed to the increased ionic mobility^14^. Above melting temperature, the enhanced conductivity is due to latter because the PVdF-HFP is in its amorphous state. This is attributed to the Lewis acid-base interaction associated with the electron acceptor character of Zr^4+^ cations and oxygen vacancies that exists on the surface of ZrO_2_ nanoparticles. The interaction of these Lewis sites with PVdF-HFP polymer and PF_6_^−^ ions enhance the Li^+^ mobility^15^. However, former plays an important role at the below the melting temperature. As seen in XRD results, the increase in wt% of ZrO_2_ decreases the crystalline phase of host PVDF-HFP polymer, thus the ionic conductivity increases correspondingly. Also, the incorporated ZrO_2_ nanoparticles as a dielectric material supplement the dissociation of LiPF_6_ and facilitate ion transport^10^. However, above the optimum concentration of 7 wt%, the ZrO_2_ nanoparticles tend to agglomerate and act as mere insulator to ionic mobility.

Figure 5b shows the temperature dependence of the ionic conductivity for esPME and 7 wt% of ZrO_2_ incorporated esCPME. At the lower temperatures, glacial behaviour of the liquid electrolyte attributed to the lower ionic conductivity. At higher temperature, conversely ionic mobility increases the ionic conductivity. As the temperature increases, the polymer produces free volume due to expansion. This leads to increased segmental mobility that will assist ions transport and virtually compensate for retarding the ion associates. The Arrhenius plot of ionic conductivity was interpreted by Vogel-Tamman-Fulcher (VTF) equation^16^:


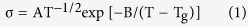


where, T_g_ is ideal glass transition temperature at which the ionic conductivity is frozen, A is pre-exponential constant related to the number of charge carriers in the electrolyte system and B is pseudo-activation energy of the ion transport. The lower effective activation energy for ions transfer at higher temperatures attributed to the increase in ionic conductivity of esCPME (7 wt% ZrO_2_)^17^. It is observed that the esPME loses its consistency above 70 °C, whereas the esCPME (7 wt% ZrO_2_) is stable and its conductivity increases with increase in the temperature up to 80 °C. It is due to the presence of ZrO_2_ nanoparticles which act as barricade for mass and heat transfer during thermal decomposition. It revealed that ZrO_2_ incorporated esCPME is suitable for wide range of temperature operation.

Figure 5c shows the linear sweep voltammograms (LSV) of esPME and 7 wt% of ZrO_2_ incorporated esCPME. The current onset voltage (anodic breakdown voltage) of esCPME (7 wt% ZrO_2_) is around 3.4 V which is higher than that of esPME (2.4 V). This is due to the interaction of Lewis acid surface of ZrO_2_ nanoparticles with Lewis base site (PF_6_^−^) of LiPF_6_ that slow down the decomposition of PF_6_^−^, thereby enhancing the electrochemical stability of esCPME^18^. The higher electrochemical stability window for esCPME (7 wt% ZrO_2_) is also be due to the increased electrolyte uptake resulting from the increase in porosity of the membrane as well as the improved electrode interfacial contact of esCPME with the electrodes^19^. Thus, the electrochemical potential window of esCPME gets improved on the incorporation of 7 wt% ZrO_2_ nanoparticles. Considering, the working voltage of Li-ion capacitor which is typically around 2.0 V to 3.0 V, the anodic breakdown voltage of esCPME (7 wt% ZrO_2_) is high enough to allow the safe use of the same for Li-ion capacitor.

**Figure 6:**
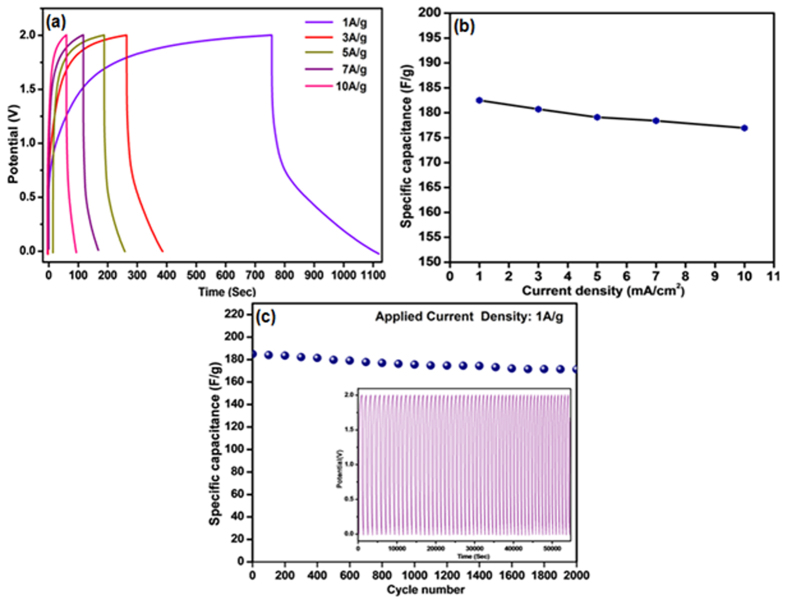
(**a**) Charge-discharge curve of Li-ion capacitor at different current densities. (**b**) Specific capacitance of Li-ion capacitor as a function of current density. (**c**) Specific capacitance as a function of charge/discharge cycles for Li-ion capacitor assembled using esCPME (7 wt% ZrO_2_).

The galvanostatic charge–discharge curves of Li-ion capacitor with 7 wt%. of ZrO_2_ incorporated esCPME at different current densities (1, 3, 5, 7 and 10 Ag^−1^) are shown in Fig. 6a. Its corresponding specific capacitance as the function of current density is shown in Fig. 6b. At a current density of 1 Ag^−1^, the capacitance of the Li-ion capacitor is calculated as high as 182.5 Fg^−1^. The specific capacitance still maintains at a high value of about 176.94 Fg^−1^ even at the high current density of 10 Ag^−1^. It depicts that the incorporation of ZrO_2_ into the esCPME has enhanced the performance of Li-ion capacitor. The increase in current density affects the Li-ion capacitor performance due to the decrease in time required for attaining the cut-off voltage.

Figure 6c shows the discharge capacity versus cycle number of Li-ion capacitors assembled with esCPME incorporated with 7 wt% of ZrO_2_. The cycling stability of Li-ion capacitor carried out by continuous charge–discharge studies at 1 Ag^−1^ for 2000 cycles. The inset is the charge-discharge curves of Li-ion capacitor at 1Ag^−1^. The Li-ion capacitor with 7 wt% of ZrO_2_ incorporated esCPME exhibits good cyclic stability even after 2000 charge–discharge cycles. It retained a high specific capacitance of 167.4 Fg^−1^ which is nearly 92% of its initial capacitance (182.5 Fg^−1^). The hexafluorophosphate system required a higher potential to charge and hence charging process is longer than the discharging process^20^. It is also observed that the Li-ion capacitor assembled with esCPME (7 wt% ZrO2) maintain nearly 99.9% of coulombic efficiency even after 2000 cycles which indicates its superior cyclic stability (Fig. S5). The improved interfacial stability of the esCPMs due to the presence of the high surface area of ZrO_2_ nanoparticles that prevent passivation, may contribute to better capacity retention.

Figure S6 shows the Ragone plot showing the dependence between power density and energy density. The power density and energy density are obtained from the galvanostatic charge-discharge curves of 7 wt% of ZrO_2_ incorporated esCPME at different current densities (1, 3, 5, 7 and 10 Ag^−1^). The obtained high energy density and power density makes it as an excellent candidate for Li-ion capacitor applications^21^.

## Conclusion

PVdF-HFP based electrospun nanocomposite polymer membrane electrolyte has been prepared by incorporating different weight percentages (0, 3, 5, 7 and 10 wt %) of ZrO_2_ nanoparticles. The incorporation of ZrO_2_ nanoparticles reduces PVdF-HFP crystallinity as evidenced from XRD and DSC studies and hence esCPMEs displayed higher ionic conductivity than esPME. The 7 wt% of ZrO_2_ incorporated esCPME offers better thermal/dimensional stability, mechanical strength and electrochemical performance than other systems. The decrease in performance beyond the optimum concentration was due to the formation of well-defined crystalline region. The temperature dependence of ionic conductivity obeys Arrhenius type activation process. The ZrO_2_ nanoparticles not only attributed to the ionic conductivity but also broad electrochemical window of 3.4 V. The Li-ion capacitor assembled with 7 wt% of ZrO_2_ incorporated esCPME exhibits specific capacitance of 182.5 Fg^−1^ at the current density of 1 Ag^−1^ and good cyclic durability with 92% capacitance retention even after 2,000 cycles. These results demonstrated the potential of 7 wt% of ZrO_2_ incorporated electrospun PVdF-HFP nanocomposite polymer membrane electrolyte for high performance Li-ion capacitors.

## Methods

### Materials

Zirconia (ZrO_2_) nanoparticles (~45 nm) were purchased from SRL. Poly [(vinylidene fluoride)-co-hexafluoropropylene] (PVDF-HFP) (Kynar flex 2801) was procured from Arkema Acetone, dimethylacetamide and Lithium hexafluorophosphate (LiPF_6_) were purchased from Sigma-Aldrich. Ethyl carbonate (EC) was procured from Alfa Aesar and Dimethyl Carbonate (DMC) from Merck. All the chemicals are of analytical grade and used without any further purification.

### Preparation of esPM and esCPMs

A homogenous 16 wt% of PVdF-HFP solution was obtained by using a solvent mixture containing acetone/N, N’-dimethyl acetamide at the weight ratio of 70/30 with a constant stirring for 12 h. Electrospinning was conducted at an applied voltage of 14 kV at the flow rate of 0.5 ml/h to collect the electrospun PVdF-HFP membrane on the grounded stainless steel substrate at a fixed distance of 12 cm from the needle tip. This was removed from the substrate and vacuum dried at 80 °C for 24 h before use. The different wt% of ZrO_2_ (0, 3, 5, 7 and 10 wt%) incorporated electrospun PVDF-HFP nanocomposite polymer membranes (esCPMs) were prepared under same experimental conditions with the addition of required wt% of ZrO_2_ to the PVdF-HFP polymer solutions.

### Characterization of esPM and esCPMs

The morphology of esPM and esCPMs were examined by using scanning electron microscopy (Hitachi, Model S-4200). The X-ray diffraction patterns of the prepared esPM and esCPM were recorded in the range of 10^ο^ to 70^ο^, with the step size and scan rate at 0.1° and 2°, respectively. (Rigaku; Model: Ultima IV). FTIR spectra were recorded in the wavenumber range of 2000 cm^−1^ to 400 cm^−1^ (Thermo Nicolet, Model: 6700). The thermal shrinkage of esPM and esCPMs was evaluated by measuring the dimensional change after being subjected to heat treatment at various temperatures for 30 min.

The crystallinity of esPM and esCPM were examined from DSC measurements in a temperature range of 30 °C to 250 °C at a heating rate of 10 °C/min (TA instrument, Model: Q600 SDT). The crystallinity of the esPM and esCPMs were calculated as follow:


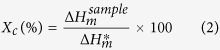


where 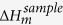 is the heat of melting of the sample and 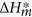 is the crystalline melting heat of PVdF, 104.7 J/g. Thermogravimetric measurements were performed in a nitrogen atmosphere from 20 to 300 °C at a rate of 10 °C min^−1^ (TA instrument, Model: Q600 SDT).

The porosity (*P*) of esCPMs was determined by weighing the membrane with and without 1-butanol from the following equation^8^:


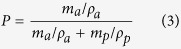


where 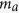 is the weight of esCPM after impregnation in 1-butanol, 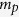 is the weight of esCPM before impregnation in 1-butanol, 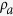 and 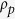 are density of 1-butanol and the dried esCPM, respectively.

To measure the electrolyte uptake of both esPM and esCPMs, the membranes were soaked in liquid electrolyte containing 1 M LiPF_6_ in EC and DMC (1:1 v/v ratio) for 24 h. They were then taken out from the liquid electrolyte solution and the excess electrolyte solution on the membrane was wiped off using Whatman filter paper. Electrolyte uptake (*U*) was estimated using the formula^9^:


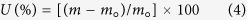


where *m* and *m*_*o*_ are the mass of wet and dry electrospun esCPMs, respectively.

The leakage of electrolyte was calculated using the equation^8^:


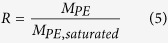


where *R* is the relative absorption ratio of liquid electrolyte, *M*_*PE, saturated*_ is the mass of esCPME when the membrane is fully saturated with the liquid electrolyte, *M*_*PE*_ is the mass of esCPME after a time interval when the saturated polymer membrane electrolyte is squeezed by pressing it between the filter papers.

The thermal stability of the polymer membranes was determined by measuring the thermal shrinkage of the membrane (separator) at various temperatures from 50 °C to 150 °C for 30 min using the equation^22^:


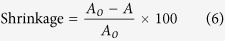


where A_o_ and A are the areas of membrane before and after the thermal treatment, respectively. The mechanical strength measurements of the esPM and esCPMs were carried out using Universal testing machine (Model: DAK SERIES 9000 (UTB - 9251)).

### Electrochemical studies

The different esCPMs were soaked in the liquid electrolyte to form their corresponding esCPMEs. The ionic conductivity (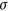) of resulting esCPMEs was measured by sandwiching the esCPME in between two stainless steel blocking electrodes and recorded their AC-impedance spectra (Biologic, Model: VSP) at 25 °C. The spectra were obtained over the frequency range of 100 KHz to 1 mHz with AC amplitude of 10 mV. The ionic conductivity of esCPME was calculated using the equation:


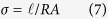


where 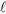 is the polymer membrane thickness, *A* is the area of the esCPME and *R* is the bulk resistance. The thickness (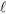) of esCPME was determined by using a digital micrometer (Mitutoyo, Japan) and was found to be 20 μm. The area (*A*) of the polymer membrane electrolyte was 1 cm^2^.

The electrochemical stability (i.e. working potential range) of the esCPME is an important parameter for the application point of view in Li-ion capacitors. Linear sweep voltammetry (LSV) was used to evaluate the working potential range of the esCPMEs by sandwiching them in between two stainless steel blocking electrodes at the scanning rate of 1 mV s^−1^ (Biologic, Model: VSP).

Li-ion capacitors were assembled by using 7 wt% of ZrO_2_ incorporated esCPME as follows; The cathode was prepared by mixing LiCo_0.2_Mn_1.8_O_4_ nanofibers, activated carbon and polyvinylidene fluoride (PVdF) in N- methyl pyrrolidone (NMP) as binder in the ratio of 85:10:5 wt%^10^ to form slurry. This slurry was coated on 2 cm^2^ stainless steel disc and dried at 80 °C for 4 hr to use as cathode. The anode was prepared by mixing 95 wt% of exfoliated activated carbon and 5 wt% of PVdF in NMP as binder. This composite mixture was coated on 2 cm^2^ stainless steel disc and dried at 80 °C for 4 hr to use as anode. Finally, a coin type Li-ion capacitor cell was assembled by using base coin as a cathode material, 7 wt% of ZrO_2_ incorporated esCPME as the electrolyte as well as separator and upper coin as an anode material. The schematic representation of assembled Li-ion capacitor cell is shown in Fig. S7.

The performance of Li-ion capacitors was investigated by a constant current charge-discharge cycling using an electrochemical analyzer (VSP, Bio-Logic, France). Galvanostatic charge-discharge curves of the Li-ion capacitor (LiCo_0.2_Mn_1.8_O_4_ /esCPME(7 wt% ZrO_2_)/Activated carbon) was carried out in the potential range of 0.0 to 2.5 V at different current densities. The specific capacitance of the electrode can be evaluated according to the following equation:


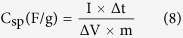


where, C_sp_ is the specific capacitance of the electrode based on the mass of active material (Fg^−1^), I is the current during discharge process, ∆t is the discharge time, ∆V is the potential window, and m is the mass of active material in the electrode.

## Additional Information

**How to cite this article:** Solarajan, A. K. *et al*. Dimensional stability and electrochemical behaviour of ZrO_2_ incorporated electrospun PVdF-HFP based nanocomposite polymer membrane electrolyte for Li-ion capacitors. *Sci. Rep.* **7**, 45390; doi: 10.1038/srep45390 (2017).

**Publisher's note:** Springer Nature remains neutral with regard to jurisdictional claims in published maps and institutional affiliations.

## Supplementary Material

Supplementary Information

## Figures and Tables

**Table 1:** Influence of different wt% of ZrO_2_ incorporated esCPMs on Porosity and electrolyte uptake and the corresponding data obtained from DSC thermograms and Nyquist plots.

Membrane	Porosity (%)	Electrolyte uptake (%)	Melting Temperature (°C)	Crystallinity (X_c_) (%)	Ionic conductivity (S/cm)
esPM	82.2	341	166.90	48.17	1.320 × 10^−3^S/cm
esCPM(3 wt% ZrO_2_)	87.9	395	166.12	45.25	2.136 × 10^−3^S/cm
esCPM(5 wt% ZrO_2_)	91.5	447	165.97	44.92	2.372 × 10^−3^S/cm
esCPM(7 wt% ZrO_2_)	95.7	481	165.54	44.25	2.695 × 10^−3^S/cm
esCPM(10 wt% ZrO_2_)	85.4	379	166.78	47.86	1.495 × 10^−3^S/cm
