# Supplementary material for: Dimensional stability and electrochemical behaviour of ZrO2 incorporated electrospun PVdF-HFP based nanocomposite polymer membrane electrolyte for Li-ion capacitors
Source: Sci Rep. 2017 Apr 11;7:45390. doi: 10.1038/srep45390 (PMC5387720; doi:10.1038/srep45390)
Supplement: Supplementary Information [file srep45390-s1.docx]

SUPPLEMENTARY INFORMATION

Dimensional stability and electrochemical behaviour of ZrO_2_ incorporated electrospun PVdF-HFP based nanocomposite polymer membrane electrolyte for Li-ion capacitors

*Arun Kumar Solarajan, Vignesh Murugadoss, Subramania Angaiah**

*Electrochemical Energy Research Lab, Centre for Nanoscience and Technology,*

*Pondicherry University, Puducherry - 605 014, India.*

*(*Corresponding Author E-mail:* [*a.subramania@gmail.com*](mailto:a.subramania@gmail.com)*)*

[*Tel:+91-413-2654980*](Tel:+91-413-2654980)*, Fax (+91)413-2655348*

**Table of Contents**

**I.** Porosity and Electrolyte uptake as a function of different wt% of ZrO_2._

**II.** DSC thermograms for different wt% of ZrO_2_ incorporated esCPMs.

**III.** TGA curve for different wt% of ZrO_2_ incorporated esCPMs.

**IV.** Thermogravimetric parameters of different wt% of ZrO_2_ incorporated esCPMs.

**V.** Ionic conductivity at different wt% of ZrO_2_ incorporated esCPMEs.

**VI.** Coulombic efficiency for Li-ion capacitor assembled using esCPME (7wt% ZrO_2_) as a function of charge/discharge cycle

**VII.** Ragone plot for Li-ion capacitor assembled using esCPME (7wt% ZrO_2_)

**VIII.** A schematic representation of assembled Li-ion capacitor coin cell.


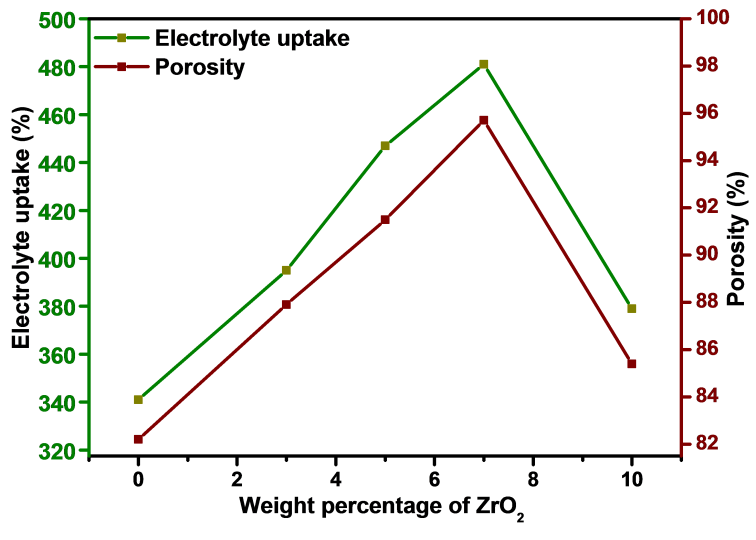


**Fig. S1** Porosity and Electrolyte uptake as a function of different wt% of ZrO_2._


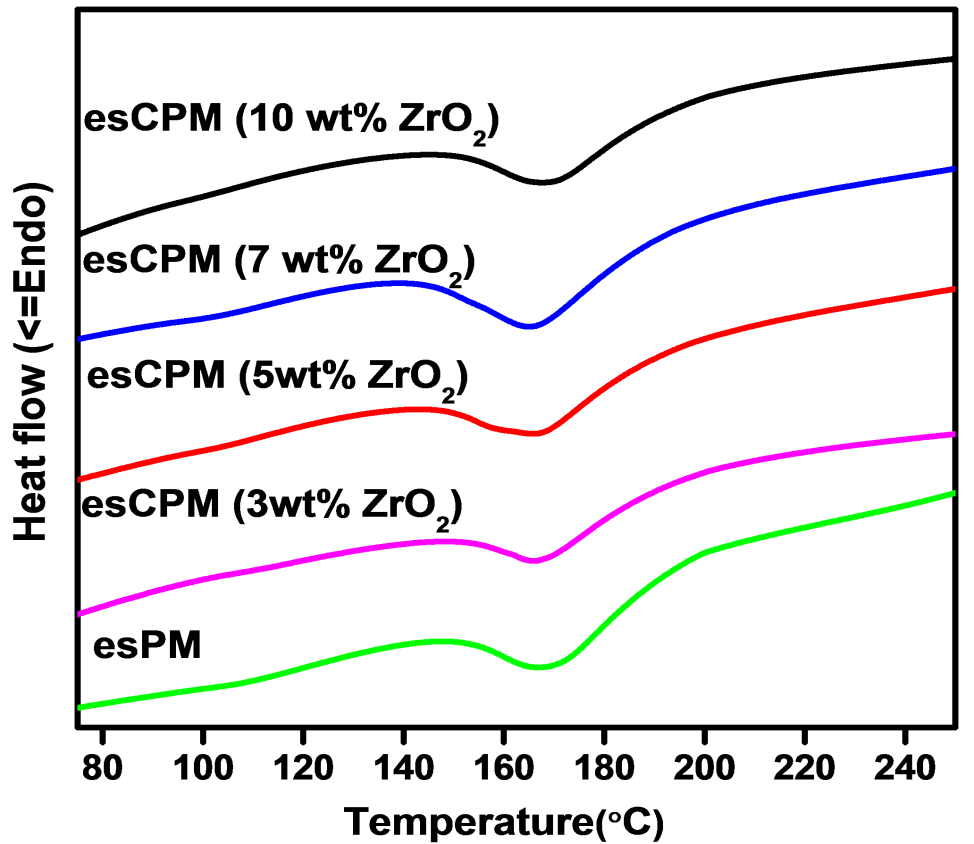


**Fig. S2** DSC thermograms for different wt% of ZrO_2_ incorporated esCPMs.


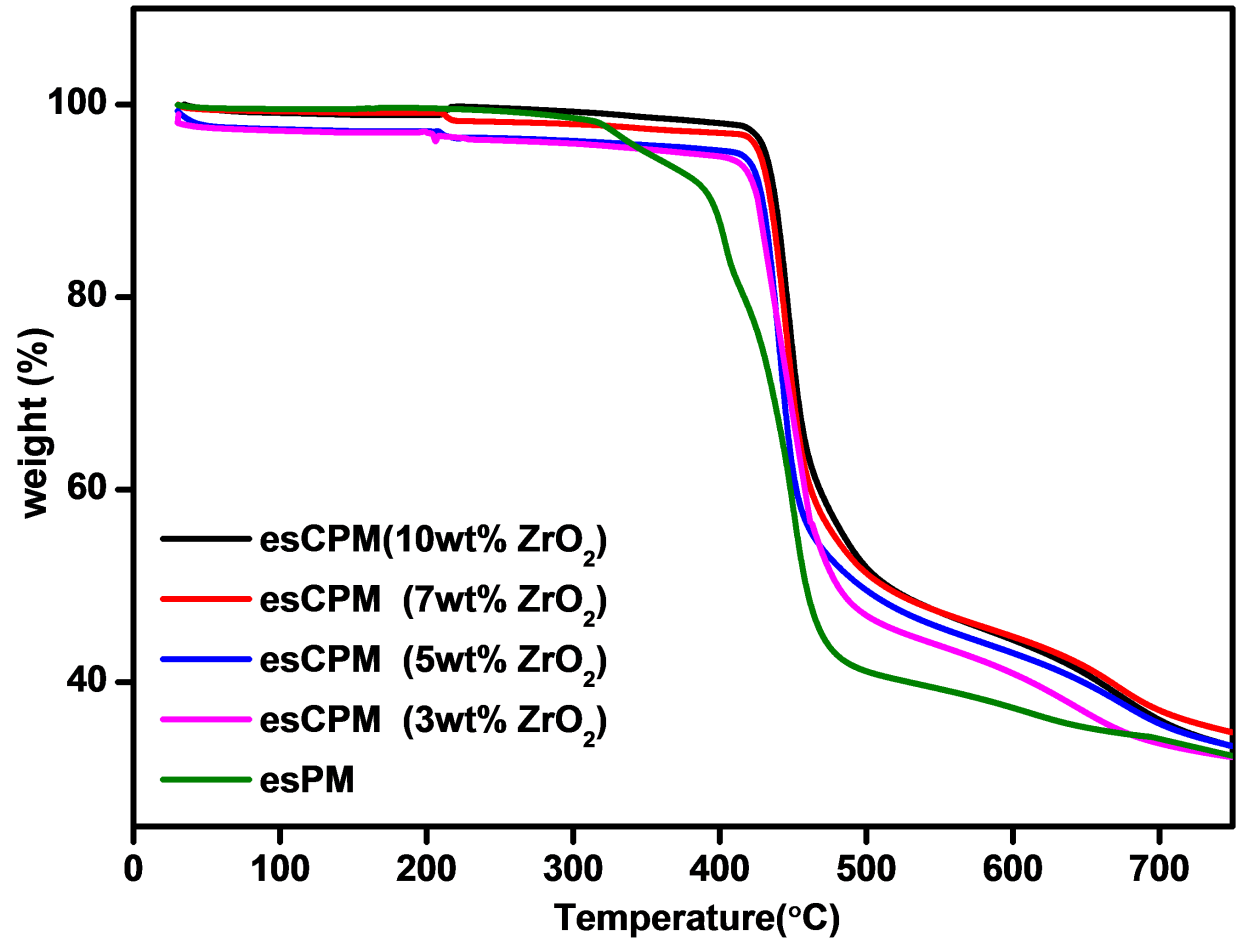


**Fig. S3** TGA curve for different wt% of ZrO_2_ incorporated esCPMs.

**Table S1.** Thermogravimetric parameters of different wt% of ZrO_2_ incorporated esCPMs.

| **Membrane** | **T_onset_ (°C)** | **T_d_**  **(°C)** | **Residue**  **(% char)** |
| --- | --- | --- | --- |
| esPM | 380.17 | 467.21 | 34.31 |
| esCPM(3wt% ZrO_2_) | 424.51 | 480.47 | 34.31 |
| esCPM(5wt% ZrO_2_) | 428.63 | 481.34 | 35.97 |
| esCPM(7wt% ZrO_2_) | 430.23 | 485.56 | 37.01 |
| esCPM(10wt% ZrO_2_) | 433.40 | 485.04 | 33.32 |


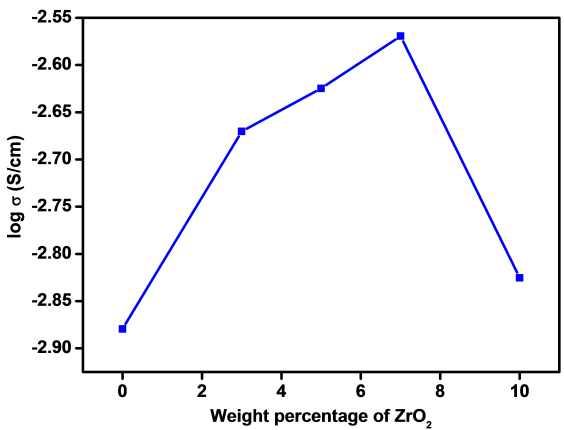


**Fig. S4** Ionic conductivity at different wt% of ZrO_2_ incorporated esCPMEs.

_
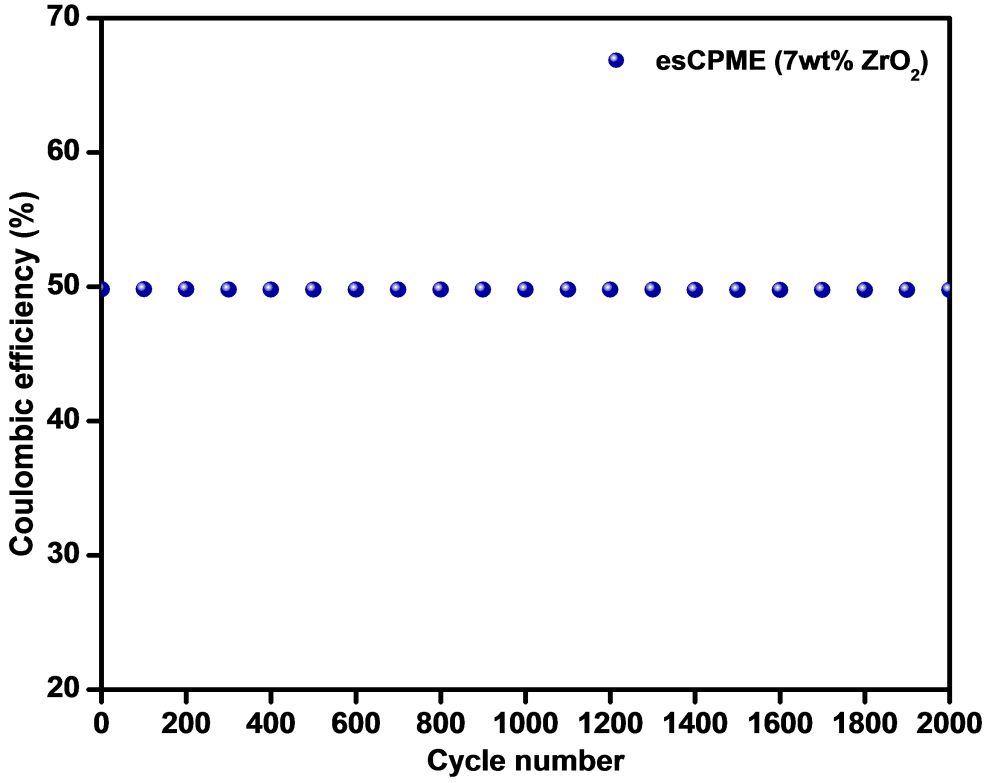
_

**Fig. S5** Coulombic efficiency of Li-ion capacitor assembled using esCPME

(7wt% ZrO_2_) as a function of charge/discharge cycle


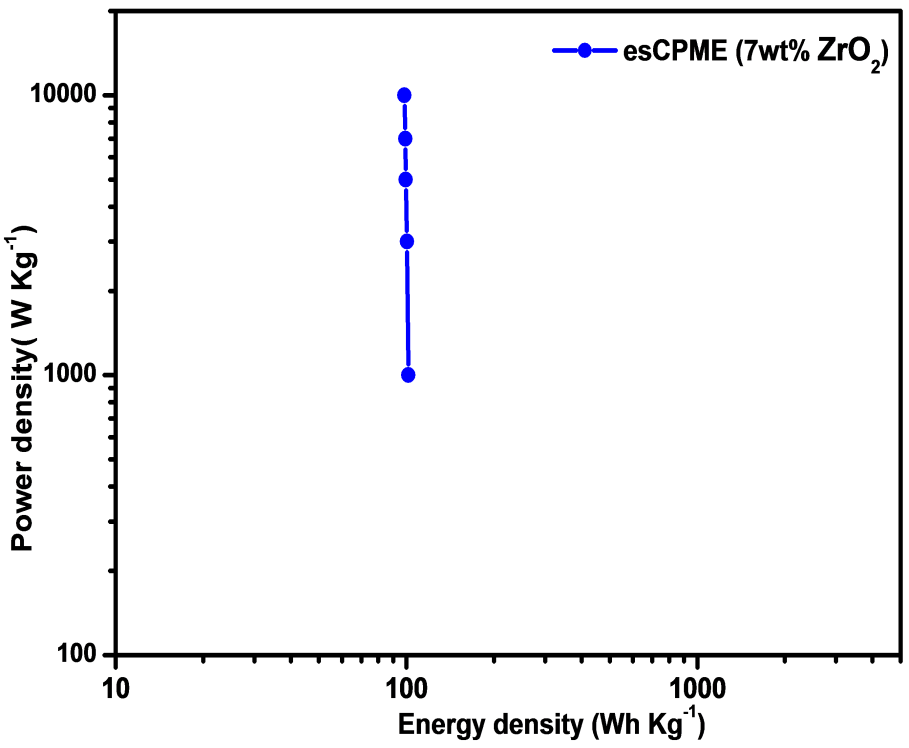


**Fig. S6** Ragone plot for Li-ion capacitor assembled using esCPME (7wt% ZrO_2_)


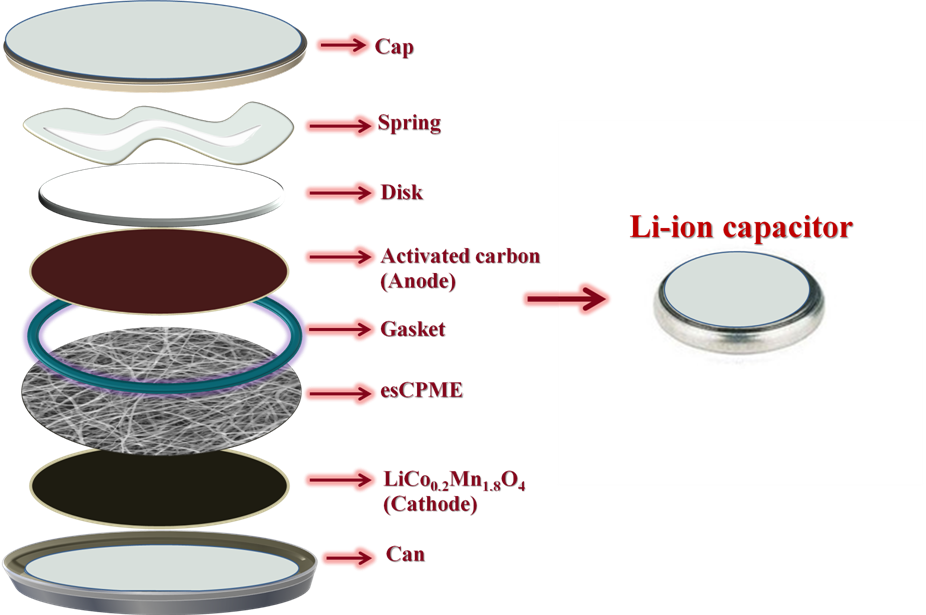


**Fig. S7** A schematic representation of assembled Li-ion capacitor coin cell.
